# Genetically Engineered *Lactococcus lactis* Protect against House Dust Mite Allergy in a BALB/c Mouse Model

**DOI:** 10.1371/journal.pone.0109461

**Published:** 2014-10-07

**Authors:** Chunqing Ai, Qiuxiang Zhang, Chengcheng Ren, Gang Wang, Xiaoming Liu, Fengwei Tian, Jianxin Zhao, Hao Zhang, Yong Q. Chen, Wei Chen

**Affiliations:** 1 State Key Laboratory of Food Science and Technology, School of Food Science and Technology, Jiangnan University, Wuxi, Jiangsu, P. R. China; 2 Synergistic Innovation Center for Food Safety and Nutrition, Jiangnan University, Wuxi, Jiangsu, P. R. China; University of Camerino, Italy

## Abstract

**Background:**

Mucosal vaccine based on lactic acid bacteria is an attractive concept for the prevention and treatment of allergic diseases, but their mechanisms of action *in vivo* are poorly understood. Therefore, we sought to investigate how recombinant major dust mite allergen Der p2-expressing *Lactococcus lactis* as a mucosal vaccine induced the immune tolerance against house dust mite allergy in a mouse model.

**Methods:**

Three strains of recombinant *L. lactis* producing Der p2 in different cell components (extracellular, intracellular and cell wall) were firstly constructed. Their prophylactic potential was evaluated in a Der p2-sensitised mouse model, and immunomodulation properties at the cellular level were determined by measuring cytokine production *in vitro*.

**Results:**

Der p2 expressed in the different recombinant *L. lactis* strains was recognized by a polyclonal anti-Der p2 antibody. Oral treatment with the recombinant *L. lactis* prior sensitization significantly prevented the development of airway inflammation in the Der p2-sensitized mice, as determined by the attenuation of inflammatory cells infiltration in the lung tissues and decrease of Th2 cytokines IL-4 and IL-5 levels in bronchoalveolar lavage. In addition, the serum allergen-specific IgE levels were significantly reduced, and the levels of IL-4 in the spleen and mesenteric lymph nodes cell cultures were also markedly decreased upon allergen stimulation in the mice fed with the recombinant *L. lactis* strains. These protective effects correlated with a significant up-regulation of regulatory T cells in the mesenteric lymph nodes.

**Conclusion:**

Oral pretreatment with live recombinant *L. lactis* prevented the development of allergen-induced airway inflammation primarily by the induction of specific mucosal immune tolerance.

## Introduction

Asthma is a chronic inflammatory disease of the airway affecting 300 million people worldwide, which is also the most common chronic disease among children [1]. Epidemiological studies have shown that despite there are geographical differences, roughly 70% to 80% of asthmatics are allergic to house dust mite (HDM) [2]. The common symptoms of asthma, including wheezing, coughing, chest tightness and shortness of breath, generally make patients uncomfortable, whereas an acute asthma exacerbation may pose a life threat to asthma patients [3]. In addition, recurrent attacks, a major characteristic of allergic asthma, have a serious effect on the quality of life in asthmatic patients. At present, 10-15% of individuals in western population have asthma; about 25% of whom experience weekly symptoms and 15% daily symptoms [4]. Attempts at HDM reduction in the management of HDM-sensitive patients are logical, but there is considerable uncertainty regarding the efficacy and effectiveness of interventions as a result of widespread existence of HDM in the environment. Therefore, more convenient and efficacious prophylactic or therapeutic strategies for HDM allergic asthma are now required.

Giving gradually increasing doses of allergen to allergic patients with the aim of inducing a state of allergen-specific unresponsiveness is a promising treatment strategy. Conventional allergen desensitization is one of the few established curative treatments for the majority of allergic diseases [5], but it is limited by the poor quality of natural allergen extracts that have been used in the production of current allergy vaccines [6]. Immunotherapy with purified recombinant allergens according to the patient's sensitization profiles may address the problem above. Pauli and colleagues demonstrated that a single recombinant allergen Bet v1 was as effective as a natural Bet v1 in the treatment of respiratory allergy [7]. At present, recombinant allergens are mainly produced in large amounts in *Escherichia coli*, yeasts or insect cell at low cost. However, the complicated purification process may limit its application in clinical treatment.

Lactic acid bacteria (LAB), widely used in the food industry for a long time, are present in the intestine of most animals including humans. Due to issues of safety and intrinsic immunomodulation properties, there has been increasing interest in the application of LAB as effective vehicles to deliver antigens or biologically active proteins in the mucosal tissues [8], [9]. The protective effects of these genetically engineered *Lactococcus lactis* strains for a wide range of diseases have been verified in a number of animal experiments and clinical trials [10]-[12]. Lee and colleagues demonstrated that recombinant Giardia lamblia cyst wall protein-2-expressing *L. lactis* significantly increased the local immune responses in the mesenteric lymph nodes and Peyer's patches, and reduced cyst output in a mouse model [10]. Furthermore, the results of clinical trials indicated that transgenic *L. lactis* expressing IL-10 can significantly reduce Crohn's disease in patients [12]. Therefore, mucosal delivery of recombinant HDM allergen-expressing *L. lactis* vaccine would be a promising approach for the immunotherapy of HDM allergic diseases. Among more than 30 HDM allergens, up to 80% of HDM-allergy patients exert positive reaction to Der p2 [13], and thus recombinant Der p2-expressing *L. lactis* could be fully efficient in the prevention and treatment of major HDM allergic diseases.

So far, several expression systems have been designed to specifically target protein or antigen to different locations (i.e., intracellular part, the cell wall or the extracellular medium) in *L. lactis*, and the final localization of heterogenous protein in recombinant LAB vaccine may influence its final immunogenicity *in vivo* [14]. There were a few researches briefly describing the influence of one or two expression patterns on the final immunogenicity of antigen-expressing mucosal vaccine *in vivo*. But the protective effect of recombinant *L. lactis* strains expressing HDM allergen via three different expression systems has not been explored. Hence, three recombinant *L. lactis* strains expressing Der p2 in the intracellular, extracellular and cell wall parts were constructed, and then the immune mechanisms involved and their prophylactic potential in mouse models were evaluated.

## Materials and Methods

### Bacterial strains, plasmids and growth condition

The bacterial strains and plasmids used in this study are listed in Table 1. *Escherichia coli* was cultured aerobically at 37°C in Luria Broth. *L. lactis* was grown at 30°C in M17 medium supplemented with glucose (0.5%). Antibiotics (Sigma, USA) were used at the following concentrations: for *E. coli*, ampicillin (100 µg/ml) and kanamycin (50 µg/ml); for *L. lactis*, chloramphenicol (10 µg/ml).

**Table 1:** Bacterial strains and plasmids used in this study.

Strains and plasmids	Characteristics	source
*L. lactis* NZ9000	*L. lactis* strain derived from *L. lactis* MG1363	In our lab
LL-E	*L. lactis* NZ9000 containing pNZ8148-ED plasmid	this work
LL-I	*L. lactis* NZ9000 containing pNZ8148-ID plasmid	this work
LL-W	*L. lactis* NZ9000 containing pNZ8148-WD plasmid	this work
*L. lactis* 8148	*L. lactis* NZ9000 containing pNZ8148 plasmid	In our lab
*E. coli* BL21(DE3)	Expression strain	In our lab
*E. coli* BL21D	*E. coli* BL21 (DE3) containing pET28a-Derp2 plasmid	this work
*E. coli* Top10	Subclone strain	In our lab
*E. coli* Top10D	*E. coli* Top10 containing pET28a-Derp2 plasmid	In our lab
**Plasmids**		
pET28a	Kan^r^, commercial expression plasmid	In our lab
pNZ8148	Cm^r^, pNZ8048 derivative; expression vector with nisA promoter	In our lab
pUC57-Derp2	Amp^r^, pUC57 plasmid carrying Derp2 gene (codon optimization)	Sangon
pET28a-Derp2	pET28a plasmid carrying Derp2 gene	this work
pNZ8148-ED	pNZ8148 carrying signal peptide of Usp45 and Der p2 gene fused to *nis*A promoter	this work
pNZ8148-ID	pNZ8148 carrying Der p2 gene fused to *nis*A promoter	this work
pNZ8148-WD	pNZ8148 carrying signal peptide of Usp45, Der p2 and fragment of CA fused to *nis*A promoter	this work

### Transformation, DNA manipulation and construction of the plasmids

To construct the *E. coli* vector, the *derp2* coding sequence from the pUC57-Derp2 plasmid (Sangon, China) was amplified using the primers 28a-DF and 28a-DR (Table 2). The resulting fragment digested by *Nhe*I and *Sac*I was cloned into a *Nhe*I-*Sac*I digested pET28a expression plasmid (named pET28a-Derp2), verified by DNA sequencing and subsequently transformed into *E. coli, yielding E. coli* BL21D.

**Table 2:** Primers used in this study.

Primers	Sequence	Restriction site
28a-DF	CGGCTAGCGATCAAGTTGATGTTAAAGATTGTGC	*Nhe*I
28a-DR	CGAGCTCTTAATCACGAATTTTAGCATGAGTAG	*Sac*I
SPusp45-F	CGAATTCCATGGTGAAAAAAAAGATTATCTCAGC	*Nco*I
SPusp45-R	GGGGTACCCAGCGTAAACACCTGACAAC	*Kpn*I
8N-F	CACCATGGATCAAGTTGATGTTAAAGATTGTGC	*Nco*I
8N-R	GCTCTAGATTAATCACGAATTTTAGCATGAGTAG	*Xba*I
8N-F'	GGGGTACCGATCAAGTTGATGTTAAAGATTGTGC	*Kpn*I
8N-R'	GCTCTAGAATCACGAATTTTAGCATGAGTAG	*Xba*I
CA-F	GCTCTAGAGACGGAGCTTCTTCAGCTGG	*Xba*I
CA-R	GGAGCTCAATAAAATAAGCATCTATG	*Sac*I

Restriction site used in cloning is underlined.

For the *L. lactis* vectors, the *derp2* fragment amplified by the primers 8N-F and 8N-R (Table 2) was digested by *Nco*I and *Xba*I, cloned into a *Nco*I-*Xba*I digested pNZ8148 expression plasmid, and introduced into *L. lactis* NZ9000 by electroporation. The resulting plasmid verified by DNA sequencing was designated pNZ8148-ID, and the obtained *L. lactis* strain was named LL-I. To construct the other two plasmids, pNZ8148-ED and pNZ8148-WD, the signal peptide of Usp_45_ and the anchor gene fragment of N-acetylmuramidase (acmA) on the *L. lactis* MG1363 genome were amplified using the respective primers [15]. Both plasmids were manipulated with a similar protocol in the correct order to obtain recombinant *L. lactis* strains LL-E and LL-W, respectively.

### Preparation of allergen Der p2 and anti-Der p2 polyclonal antibody

The expression and purification of Der p2 in the recombinant *E. coli* BL21D was performed as previously detailed [16]. The concentration of purified Der p2 was measured with a BCA protein assay kit (Pierce, USA). The purified Der p2 was used as an allergen in the mouse model and to prepare the polyclonal anti-Der p2 antibody (AbMax Biotechnology Co., Ltd., China) for Western blot analysis.

### Expression and detection of Der p2 production

For the induction of Der p2, three recombinant *L. lactis* strains (LL-I, LL-W, LL-E) were cultured to an OD_600_ of 0.5, to which 10 ng/ml of nisin was added. After 6 h of induction, the culture supernatants and bacterial cells were separated and treated as previously described [17], [18]. For the Western blot analysis, the protein samples were separated by SDS-PAGE, transferred to a poiyvinylidene fluoride membrane by electroblotting and detected with the rabbit polyclone anti-Der p 2 antibody (1∶1000) followed by a horseradish peroxidase (HRP)-conjugated goat anti-rabbit antibody (1∶10 000).

### Preparation of fresh bacterial suspensions

All bacterial suspensions we used in mice immunization protocol were freshly prepared. Before the animal experiment, the linear relation between the optical density (OD_600_) and bacterial count has been determined. Recombinant strains LL-E, LL-I, LL-W and control strain *L. lactis* 8148 were induced as described above and the bacterial cells were harvested and washed twice with sterile PBS. Then the bacterial cells were resuspended in sterile PBS to an OD_600_ of 0.7∼0.8, and total bacterial count was determined based on the linear relation. At last, all bacterial suspensions were adjusted to achieve a final concentration of 1 × 10^10^ CFU/ml in sterile PBS.

### Animal

Female BALB/c mice (SPF, 4 weeks) were purchased from Shanghai SLAC Laboratory Animals Co., Ltd. (China) and housed at the Laboratory Animals Center of Jiangnan University in a barrier environment. Mice were kept at a constant temperature of 23 ± 1°C, relative humidity of 55 ± 5% and under a regular cycle (light: dark =  12 h ∶ 12 h). The food and sterile water were given ad libitum. Ninety mice were divided randomly into six groups, and mice in each group were assigned to three cages (5 mice per cage). All mice were housed in the standard cages for one week before the experiments began.

### Ethics statement

This study was carried out in strict accordance with the European Community guidelines (Directive 2010/63/EU) for the care and use of experimental animals. The protocol was approved by the Animal Ethics Committee of Jiangnan University, China (JN No. 20121203-0120[29]). All mice were sacrificed by cervical dislocation with sodium pentobarbital anesthesia, and all efforts were made to minimize suffering.

### Immunization protocol

**Figure 1:**
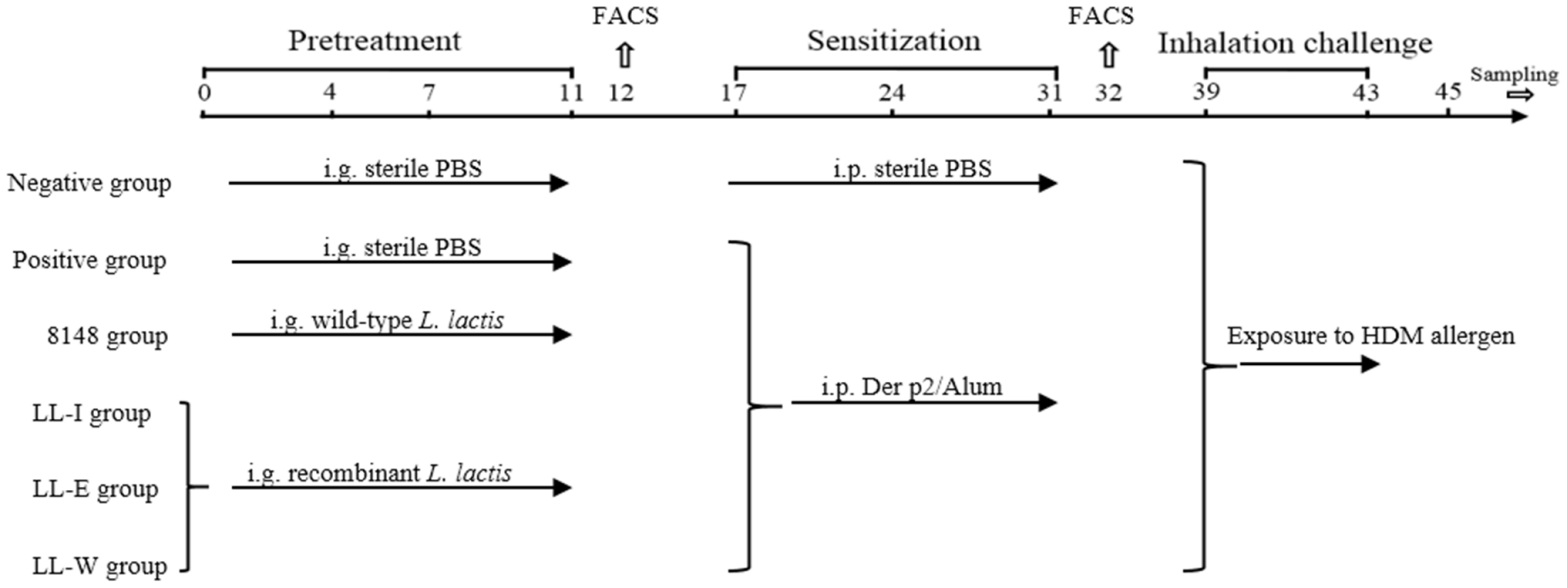
Experimental design. On days 0–4 and 7–11, BALB/c mice were daily received PBS, recombinant or wild-type *L. lactis* via the oral route. On days 17, 24 and 31, mice underwent intraperitoneal (i.p.) injection sensitization with the mixture of Der p2/Alum or sterile PBS. From day 39 to 43, all groups were inhalation challenged with aerosolized HDM allergen over a 45-min period. Positive and Negative group served as positive and negative control in this study, respectively.

The mouse models were established as described by Rigaux et al. [19]. Groups of mice (n = 15) were orally administered on days 0–4 and 7–11 with 2 × 10^9^ CFU (200 µl) of wild-type or recombinant *L. lactis*, and PBS. Seven days after the last vaccination, the mice underwent intraperitoneal injection sensitization for 3 weeks at weekly intervals with 10 µg of the purified Der p2 formulated with 2 mg of Alum (Pierce, USA). To induce airway inflammation, the sensitized mice were inhalation challenged on days 39–43 with aerosolized HDM allergen (10 mg/100 ml PBS) over a 45-min period (Fig. 1). Mice in the positive group were sensitized to Der p2, expressing significant allergic responses, and the naive mice with PBS treatment were used as a negative control.

### Measurement of specific antibodies and total IgE in serum

The levels of Der p2-specific IgE, IgG1 and IgG2a in serum were measured as previously detailed by Lee et al. [20]. In addition, the concentration of total IgE in serum was also measured by ELISA in accordance with the manufacturer's instructions (Rapidbio Lab, Langka Trade Co., Ltd., Shanghai, China).

### *In vitro* stimulation of spleen and mesenteric lymph nodes cells with Der p2

The cytokine production on the *in vitro* Der p2-stimulation of pooled spleen or mesenteric lymph nodes (MLN) cells cultures was assayed following the method detailed elsewhere [20]. Briefly, single-cell suspensions were re-suspended in RPMI1640 medium containing 10% FBS supplemented with 1% penicillin/streptomycin and 1% glutamine (Hyclone, USA). After that, 2×10^6^ cells (200 µl) were added and incubated in 96-well plates (Corning, USA) with/without Der p2 (10 µg/well) for 72 h at 37°C. The levels of IL-4, IL-5, IL-10, IL-12 and IFN-γ in the cell culture supernatants were measured by ELISA according to the manufacturer's instructions (Rapidbio Lab, Langka Trade Co., Ltd., Shanghai, China).

### Bronchoalveolar lavage

The analysis of bronchoalveolar lavage was performed as an earlier description [20]. Briefly, bronchoalveolar lavage was performed using 0.8 ml HBSS instilled bilaterally with a syringe. The bronchoalveolar lavage fluid (BALF) was collected three times by gentle aspiration and then centrifuged. The levels of IL-4, IL-5 and IL-10 in BALF were measured by ELISA in accordance with the manufacturer's instructions (Abcam, USA).

### Lung histology

The lungs were fixed with 10% PBS-buffered formalin overnight and embedded in paraffin. The fixed and embedded tissues were stained with hematoxylin and eosin (H&E) for histologic assessment using a light microscope (Leica, Germany).

### Flow cytometry analysis

Fresh spleen and MLN from different groups of mice were harvested, pooled and treated as previously described [20]. Regulatory T cells were stained with a Mouse Regulatory T cell Staining Kit in accordance with the manufacturer's instructions (eBioscience, USA). Finally, staining T cells was analyzed by FACSCaliber (BD Bioscience, USA).

### Statistical analysis

The data were expressed as means ± standard error of the mean (SEM). Statistical analysis of the results was performed using SPSS 19.0 for Windows software (SPSS Inc., USA). Statistical significances between groups were determined by one-way analysis of variance (ANOVA) followed by Duncan's test. P <0.05 was considered to be statistically significant.

## Results

### Purification of recombinant Der p2 in *E. coli* and preparation of anti-Der p2 polyclonal antibody

**Figure 2:**
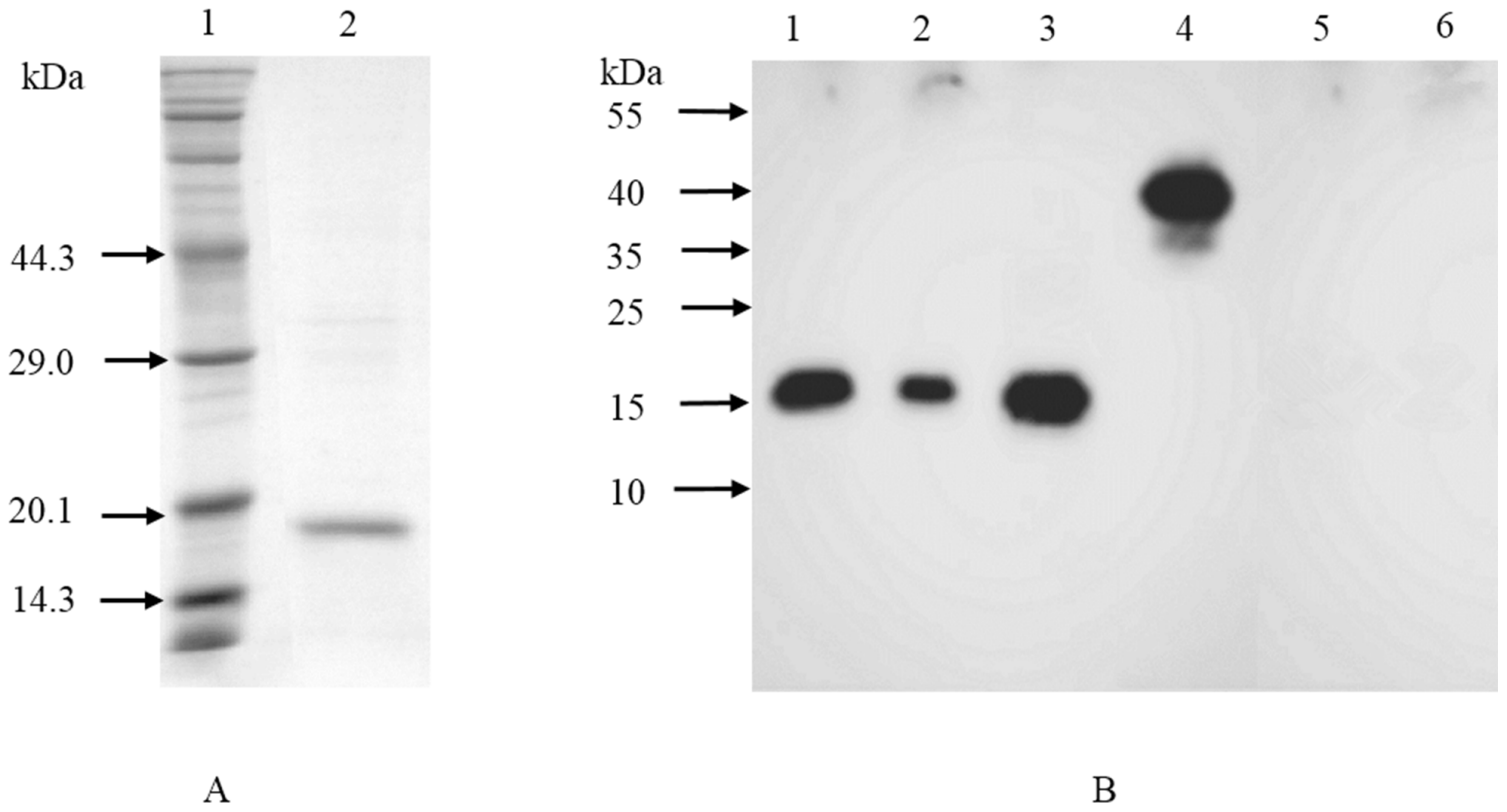
SDS-PAGE analysis of purified Der p2 from the recombinant *E. coli* (A). Lane 1: protein marker, Lane 2: purified Der p2. **Western blot analysis of recombinant Der p2-producing** ***L. lactis*** **strains** (B). Lane 1: purified Der p2 from recombinant *E. coli*, Lane 2: the culture from strain LL-E, Lane 3: the cell extract of strain LL-I, Lane 4: the cell wall extract of strain LL-W, Lane 5, 6: the culture or cell extract from wild-type *L. lactis*.

To prepare the Der p2-specific polyclonal antibody, recombinant Der p2 in *E. coli* BL21D was purified by passing a cellular extract over a nickel affinity column in accordance with the protocols from the Novagen pET system manual. The protein obtained was analyzed by SDS-PAGE (Fig. 2A). The Der p2-specific polyclonal antibody was obtained by immunizing rabbits with purified Der p2, and the final antibody titer was about 1: 1 × 10^5^.

### Production and identification of recombinant Der p2 in *L. lactis*

As the localization of an antigen in recombinant LAB strain is anticipated to influence its final immunogenicity *in vivo*, three recombinant *L. lactis* strains were engineered to express Der p2 in the extracellular environment (LL-E), intracellular (LL-I) and cell wall parts (LL-W). The production of Der p2 in the recombinant *L. lactis* was evaluated by western blot using the anti-Der p2 polyclonal antibody. After nisin induction, a band of ∼17 kDa similar to the size of purified Der p2 was detected in the culture supernatant of strain LL-E and the intracellular fraction of strain LL-I, respectively (Fig. 2B). In addition, a band of ∼40 kDa was observed in the cell wall fraction of strain LL-W corresponding to a fusion protein of Der p2 and a fragment of acmA. No signal was detected in the culture supernatant and cell extract of wild-type *L. lactis* 8148 (Fig. 2B). These results indicated that three recombinant strains successfully produced Der p2 in different cell components (extracellular environment, intracellular part, cell wall), respectively.

### Recombinant *L. lactis* strains modulated Der p2-specific antibody responses

**Figure 3:**
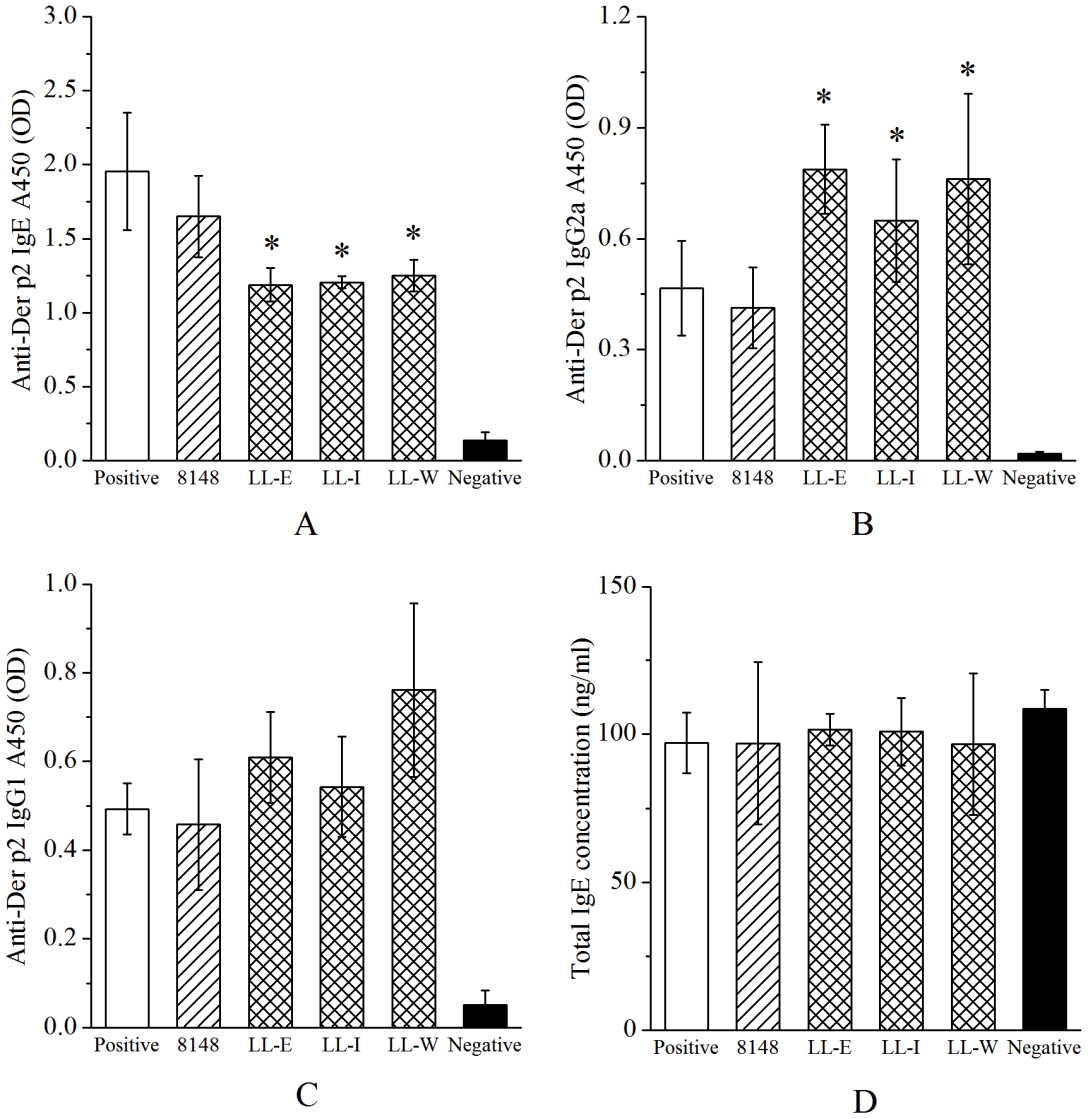
Der p2-specific antibodies and total IgE in serum were detected after inhalation challenge. Der p2-specific IgE (A), IgG2a (B) and IgG1 (C) were measured as optical density (OD) units, and total IgE (D) was reported as ng/ml. Results are means ± SEM. ^*^P <0.05 vs the positive group.

Aiming at evaluating whether the treatment with the recombinant *L. lactis* influenced systemic antibody responses, the levels of Der p2-specific antibody (IgE, IgG1, IgG2a) and total IgE in serum were measured. The Der p2-sensitized mice developed apparent Th2-biased specific antibody responses characterized by high levels of specific IgE and IgG1, whereas the treatment with any recombinant *L. lactis* significantly reduced the specific IgE responses and increased the specific IgG2a levels in serum compared with the positive group (Fig. 3A and B). It seemed that there was more specific IgG2a production in the mice fed with strain LL-E and LL-W than in those fed with strain LL-I. However, this immunomodulation was not observed in those mice fed with wild-type *L. lactis* 8148 (Fig. 3A and B). Moreover, the levels of specific IgG1 and total IgE were not modulated by the recombinant or wild-type *L. lactis* (Fig. 3C and D), and no significant signal was detected in the negative group.

### Recombinant *L. lactis* strains suppressed the *in vitro* cellular responses

**Figure 4:**
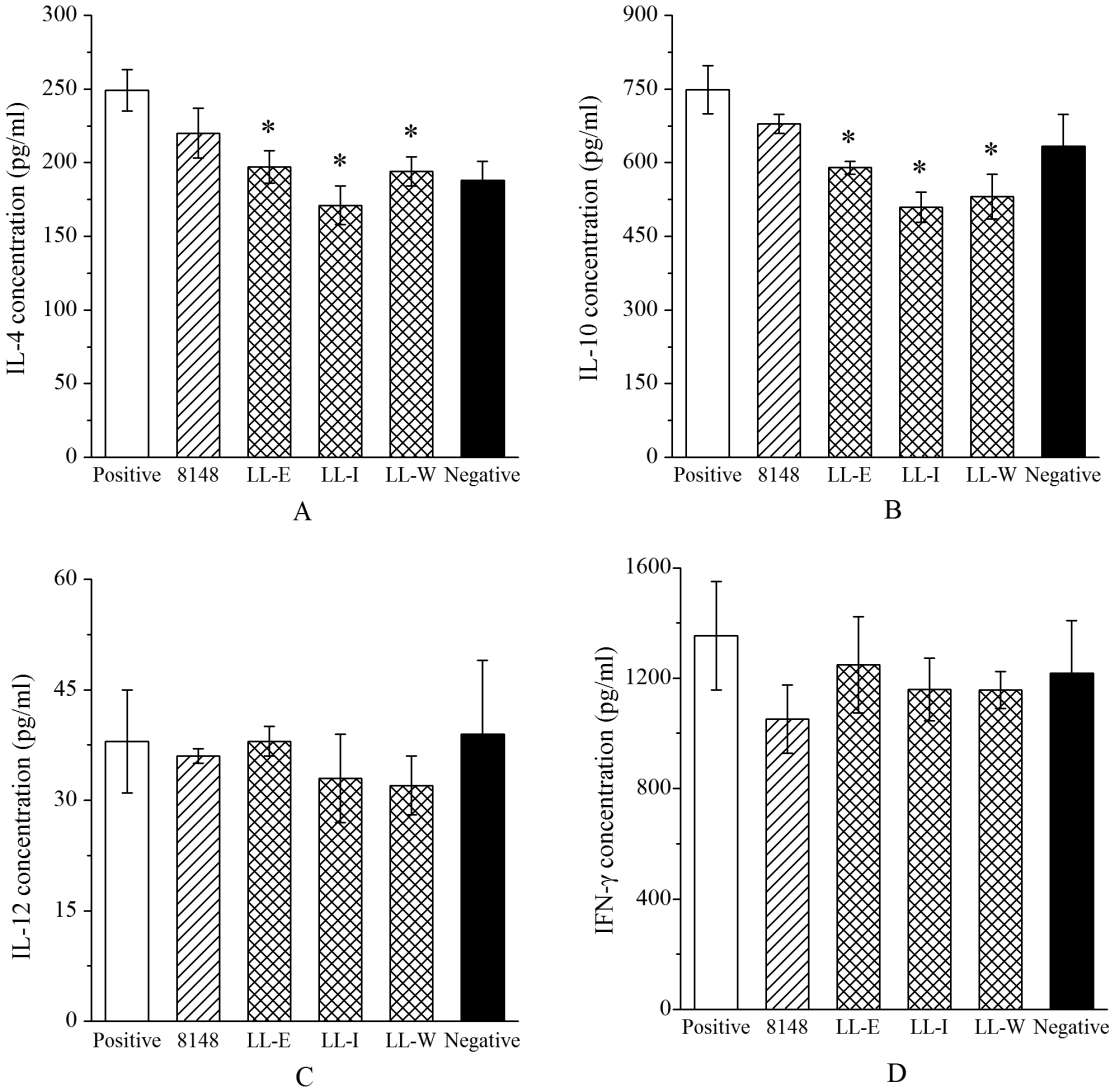
Effect of oral administration with the recombinant *L. lactis* on cytokine production. After *in vitro* stimulation with Der p2, the levels of IL-4 (A), IL-10 (B), IL-12 (C) and IFN-γ (D) in the supernatants of spleen cells from the respective groups were measured. Results are expressed as means ± SEM. ^*^P <0.05 vs the positive group.

To further evaluate the effect of the recombinant *L. lactis* on the systemic and mucosal immune response to allergen Der p2, the levels of cytokines in the Der p2-stimulated spleen and MLN cell cultures were measured. In the spleen cell cultures, the treatment with both recombinant and wild-type *L. lactis* resulted in a marked reduction in IL-4 and IL-10 levels relative to the positive group, whereas the reduction was most pronounced in the mice immunized with three recombinant strains (Fig. 4A and B). No significant effects on IFN-γ and IL-12 levels were observed in all pretreated groups (Fig. 4C and D). In addition, IL-5 production was not detectable in the spleen cell cultures (data not shown). In the MLN cell cultures, IL-4 and IL-10 production were also significantly suppressed in the mice fed with any recombinant *L. lactis* compared with the positive group or group treated with wild-type *L. lactis*. Levels of cytokines IFN-γ, IL-12 and IL-5 were not modulated by the recombinant or wild-type *L. lactis* in the Der p2-sensitized mice (Table 3).

**Table 3:** Cytokine production in the MLN cell culture supernatants *in vitro*.

Groups	Cytokine (pg/ml)				
	IFN-γ	IL-10	IL-4	IL-12	IL-5
Positive	1327±170	655±31	236±16	38±6	3.6±0.2
8148	1286±192	508±41^*^	214±27	37±1	2.6±0.2
LL-I	1300±180	505±28^*^	166±12^*^	33±6	2.6±0.2
LL-W	1270±178	496±18^*^	178±19^*^	31±5	3.1±0.4
LL-E	1317±95	531±40^*^	157±36^*^	36±3	3.6±0.3
Negative	1298±57	584±17	216±14	37±4	3.3±0.3

Results were expressed as means ± SEM, ^*^P <0.05 compared with the positive group.

### Recombinant *L. lactis* strains alleviated pulmonary inflammation

**Figure 5:**
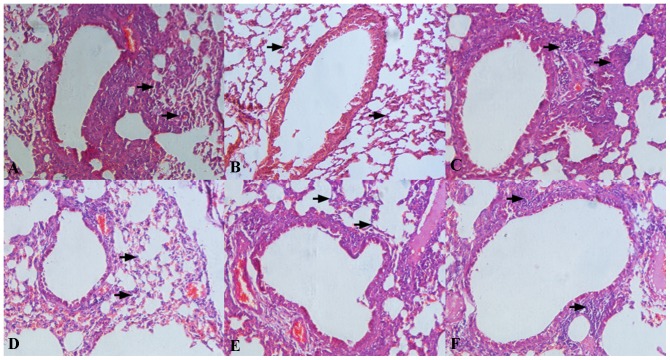
Histological analysis. Representative images (200x) showed the extensive infiltration of inflammatory cells in the lung tissues from the respective groups: Positive group (A), Negative group (B), *L. lactis* 8148 (C), LL-E (D), LL-I (E), LL-W (F). Inflammatory cells were indicated with black arrows.

To investigate whether the recombinant *L. lactis* suppressed Der p2-induced airway inflammation, histological analysis of the lung tissues and cytokine production in bronchoalveolar lavage fluid (BALF) were investigated. After the inhalation challenge, the examination of the lung tissues from the positive group revealed numerous inflammatory cells surrounding the airways, whereas the treatment with any recombinant *L. lactis* produced a marked decrease in both cellular infiltration and inflammatory changes similar to the negative group, as determined by histopathology (Fig. 5). Oral treatment with both recombinant and wild-type *L. lactis* significantly decreased Th2 cytokines IL-4 and IL-5 content in BALF compared with the positive group, whereas the reduction was most pronounced in the mice fed with three recombinant strains. Surprisingly, IL-10 levels were not affected by any of the *L. lactis* strains (Table 4).

**Table 4:** Cytokine production in bronchoalveolar lavage fluid.

Group	IL-4 (pg/ml)	IL-5 (pg/ml)	IL-10 (pg/ml)
Positive	98 ± 13	174 ± 20	375 ± 27
8148	73 ± 6*	134 ± 6*	340 ± 14
LL-I	56 ± 13*	97 ± 5* ^#^	348 ± 18
LL-W	60 ± 6*	93 ± 11* ^#^	313 ± 12
LL-E	63 ± 7*	88 ± 7* ^#^	302 ± 14
Negative	27 ± 4	33 ± 7	304 ± 47

Results were reported as means ± SEM.

*P <0.05 compared with the positive group; ^#^P <0.05 compared with the 8148 group.

### Recombinant *L. lactis* strains induced the production of Tregs in the MLN

**Figure 6:**
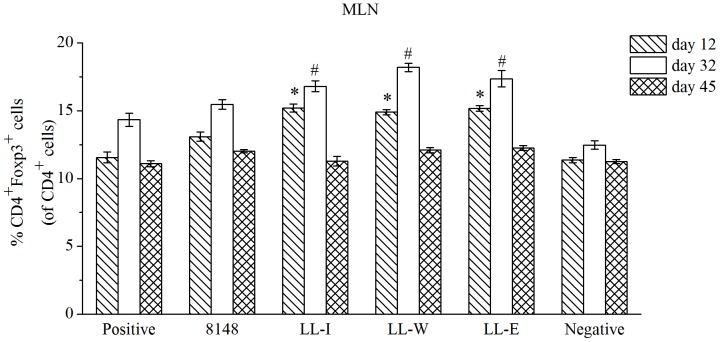
Effect of the recombinant *L. lactis* treatment on the proportion of CD4^+^Foxp3^+^ T cells in the MLN. On days 12, 32 and 45, the levels of CD4^+^Foxp3^+^ T cells in the MLN from the respective groups were analyzed by FACS. Results were reported as means ± SEM. ^*^P <0.05 vs the positive group (day 12). ^#^P <0.05 vs the positive group (day 32).

To investigate how the recombinant *L. lactis* modulated the intestinal mucosal immune responses to inhibit allergic responses, the percentage of CD4^+^Foxp3^+^ T cells in the MLN was analyzed. After oral treatment with the recombinant or wild-type *L. lactis* for a short time, the levels of Tregs were significantly increased relative to the positive group, whereas three recombinant strains had more significant effect on the Tregs levels than wild-type *L. lactis* (Fig. 6, day 12). When the mice were sensitized to Der p2, the treatment with any recombinant *L. lactis* seemed to be superior to those with wild-type *L. lactis*, inducing a significant increase in the Tregs level (Fig. 6, day 32). After the inhalation challenge, the Tregs level in all the sensitized mice returned to a normal state similar to those in the non-sensitized mice, and there were no significant differences between the different treatment groups (Fig. 6, day 45).

### Recombinant *L. lactis* had no significant effect on the Tregs level in the spleen

**Figure 7:**
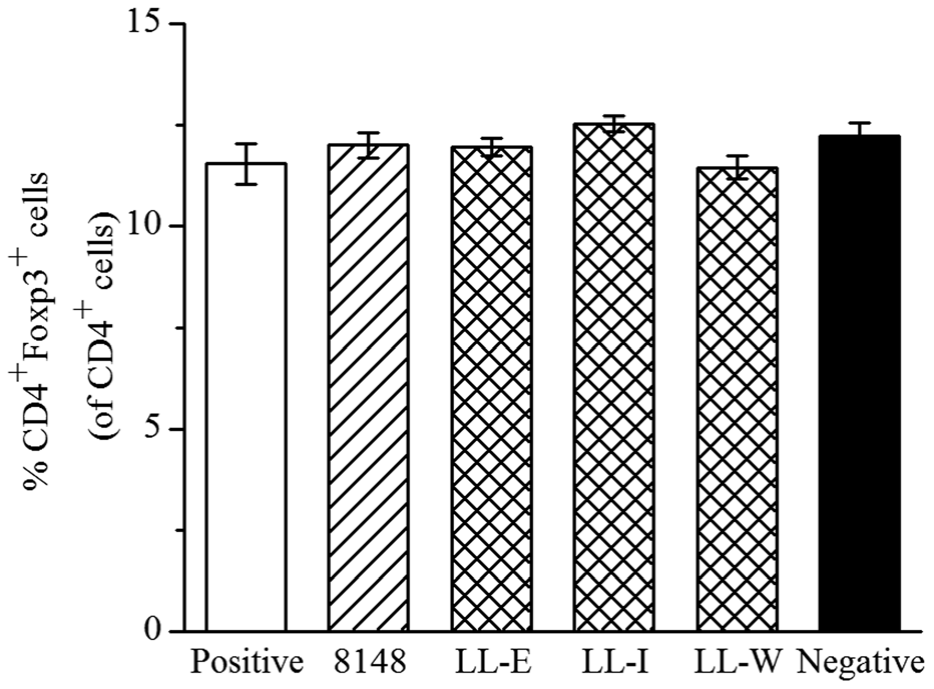
Measurement of CD4^+^Foxp3^+^ T cells in the spleen. On day 12, the percentage of CD4^+^Foxp3^+^ T cells in the spleen from the respective groups were evaluated. Results were expressed as means ± SEM.

To investigate whether the recombinant *L. lactis* modulated systemic Tregs responses to inhibit allergic responses, the levels of Tregs in the spleen were assessed. This result showed that wild-type *L. lactis* had no significant effect on the proportion of Tregs in the spleen compared with the positive group, and the same result was also observed in the mice immunized with any recombinant *L. lactis* (Fig. 7).

## Discussion

Based on the safety and immunomodulation properties, LAB have been demonstrated to act as potent mucosal adjuvants and/or antigen-delivery systems [21]. In light of this, recombinant LAB vaccine, genetically engineered to produce and deliver an allergen to the mucosal surface with the aim of inducing allergen-specific immune tolerance, has emerged as a promising concept for mucosal intervention against Type I allergy [22], [23]. Therefore, we constructed three strains of recombinant *L. lactis* producing Der p2 in different cell components, and evaluated their prophylactic potential in the mouse models.

It was assumed that the localization of an antigen in recombinant LAB vaccine is anticipated to influence its final immunogenicity *in vivo* [24]. In this study, there was no statistically significant difference among different recombinant strains in the suppression of allergic responses as a whole. These results were different from that of a previous study, demonstrating that mice immunized with cell-wall-anchored E7 antigen exerted a better protective effect than those immunized with a strain expressing intracellular E7 antigen [25]. Although it may indicate that the cell-wall-anchored form of an antigen is more immunogenic, it could also be due to the production of increased total amount of antigen by recombinant LAB strain. This can be explained by the concept that cytoplasmic proteolysis of expressed proteins or the toxicity of heterologous proteins to the host strains can be avoided if the synthesized proteins are transported extracellular environment [26]. However, not all of recombinant antigen-expressing LAB vaccines exerted the same result. Norton and colleagues showed that tetanus toxin fragment C located on the cytoplasmic was more immunogenic than the cell surface antigen [27]. This may attribute to the fact that intracellular protein is avoided from proteolysis in the gastrointestinal tract and generally secreted as soluble molecules once the host strains lysed. These results showed that the best location of expressed protein for optimal mucosal immunization may be closely associated with specific properties of expressed protein, and thus cannot yet be conclusively identified. In this study, the localization of allergen Der p2 in the recombinant *L. lactis* has no significant influence on its final immunogenicity *in vivo*.

Allergen-specific IgE is usually known as a surrogate marker for the clinical diagnosis of allergic diseases [28]. The present study showed that oral prophylactic vaccination with the recombinant *L. lactis* inhibited the development of Der p2-specific allergic responses as evidenced by reduced specific IgE levels, which were closely associated with increased specific IgG2a levels. Rigaux and colleagues showed the same results that recombinant allergen-producing *L. plantarum* impaired allergic responses by increased specific IgG2a production [19]. The Th2-biased allergic immune response is indeed prevented by the induction of a specific Th1 profile characterized by a high specific IgG2a concentration. Despite the fact that the blocking activity of specific IgG2a remains to be elucidated, it is tempting to speculate that specific IgG2a could compete with specific IgE binding with Der p2 to alleviate allergic inflammation [29]. The antibody isotype switch bias to IgG2a may confer a protection against allergic symptoms in specific immunotherapy, as confirmed by Rupa and Mine [30], and thus recombinant LAB vaccine would be an effective strategy in the treatment and prevention of allergic diseases.

It has been well established that the internal profile of Th1/Th2 responses decides the orientation of antibody response [31], [32]. At the cellular level, the treatment with the recombinant *L. lactis* seemed to be superior to that with wild-type *L. lactis*, inducing a significant suppression of Th2 cytokine IL-4 in the spleen and MLN cell cultures. IL-4 has been shown to play an important role in promoting B cell proliferation and provoking IgE secretion from B cells [33]. Previous research demonstrated that IL-10, an important regulatory cytokine, is thought to be involved in several regulatory mechanisms including suppression of IgE and monitor the balance of Th1/Th2 responses [34]. Surprisingly, IL-10 levels were significantly reduced in all the pretreated groups. These results were consistent with Schwarzer et al. [9] who found that treatment with recombinant Bet v1-producing *L. plantarum* significantly reduced the levels of IL-4 and IL-10 *in vitro*. It is postulated that IL-10 could be produced as a part of a compensatory mechanism to control Th2-associated allergic responses, and may be reduced following recombinant *L. lactis* treatment if there is associated with the attenuation of allergic responses.

Allergic asthma is characterized as a chronic airway inflammation disease, and it is well recognized that a variety of inflammatory cells play a key role in this process [35]. After the inhalation challenge, inflammatory cells migrate from the peripheral blood to the inflamed sites in the airway, and Th2 cytokines are dominantly detected in BALF [36]. Under microscope, it was shown that the recombinant *L. lactis* exerted a beneficial effect on suppressing inflammatory cells infiltration in the lung tissues. Such suppressive effect was associated with the reduction in Th2 cytokines IL-4 and IL-5 in BALF, which were considered to play an important role in eosinophilic infiltration [37]. IL-10 has been reported to reduce Th2 cytokines production and eosinophilic infiltration in inflamed tissues [38], whereas no obvious effect on IL-10 production was observed in the mice fed with recombinant or wild-type *L. lactis*. Karimi and colleagues also demonstrated that *L. reuteri*-induced attenuation of the allergic airway response was closely correlated with decreased IL-5 level in BALF, not increased IL-10 secretion [39]. Accordingly, it can be postulated that the protective effect of recombinant *L. lactis* in inflamed tissues are possibly dependent primarily on the induction of immune tolerance, which can effectively inhibit allergen-induced Th2 responses.

Exquisitely balanced control mechanisms operating at mucosal sites are able to accommodate potent immune defense to prevent inflammatory responses caused by environmental allergens [40]. Prominent among multiple regulatory factors operating at the mucosal surface are diverse populations of Tregs. Josefowicz and colleagues have demonstrated that Tregs in the MLN are essential for mucosal tolerance, which is reported to play an important role in maintaining functional tolerance and regulating Th2 responses to allergen [41]-[43]. Here, we showed that the treatment with any recombinant *L. lactis* significantly increased the proportion of CD4^+^Foxp3^+^ Tregs in the MLN of non-sensitized mice (day 12). Such increase is thought to play a critical role in the intervention of the early development of allergic diseases, as demonstrated by Earle and colleagues [44] who found that Tregs could suppress the proliferation and cytokine secretion of effector T cells. Moreover, a population of Tregs could create a regulatory milieu that promotes the outgrowth of a new population of Tregs with antigen specificities distinct from those of the original Tregs population [45]. Therefore, we postulated that the treatment with any recombinant *L. lactis* would be superior to that with wild-type *L. lactis*, inducing a significant increase in the proportion of Tregs in the MLN of the Der p2-sensitized mice, and subsequent results confirmed our assumption (Fig. 6, day 32). However, it was remarkable that after the inhalation challenge, the levels of Tregs in the MLN of the Der p2-sensitized mice were returned to a normal state similar to those of the non-sensitized mice (Fig. 6, day 45). These results can be due to that Tregs in other immune organs could migrate to and remain in inflamed tissues, which play an essential role for their function *in vivo* [46]. It can also be explained by postulating that the maintenance of mucosal Tregs is linked to continuing antigen exposure, and antigen withdrawal results in rapid return to baseline Tregs numbers in the mucosa [47]. Changes of Tregs level in the MLN indicated that oral administration with probiotic or recombinant LAB vaccine can strengthen immune tolerance to suppress the early development of allergic diseases, but these protective effect is not durable and to be weakened or disappear without continuous application.

Eyles and colleagues have well described that the modulation of systemic immune responses by mucosal vaccination is developed through antigen transportation to systemic lymphoid organs (spleen) preferentially via the mucosal lymphoid tissues [48]. However, treatment with recombinant or wild-type *L. lactis* did not significantly influence the proportion of Tregs in the spleen. This result differed from Karimi et al. [39] who found that oral treatment with *L. reuteri* increased the Tregs population in the spleen, which is correlated with the attenuation of allergen-induced airway inflammation. The implication of these findings is that there are clear strain-specific immune-regulatory properties of LAB species *in vivo* [49] and the intrinsic immunogenicity of bacterial vehicle for vaccine delivery would influence the final immune response to recombinant LAB vaccine *in vivo*.

On the whole, oral treatment with the recombinant *L. lactis* prior sensitization exerted significant beneficial effects on the suppression of allergic responses, suggesting that recombinant LAB vaccine would be a desirable competitor for the prevention and treatment of allergic diseases in the future. The protective effect is mainly attributed to the enhancement of mucosal immune tolerance, which plays a critical role in the reduction of inflammatory factors (Th2 cytokines, specific IgE and inflammatory cells infiltration) both at the systemic levels and the local sites. In the current study, the localization of Der p2 in the recombinant *L. lactis* did not affect its final immunogenicity *in vivo*, whereas for its application in clinical treatment, it is necessary to further confirm the effect of other factors, i.e., inoculation route and bacterial vehicle, on the suppression of HDM allergic diseases.
